# The Divergent Function of Androgen Receptor in Breast Cancer; Analysis of Steroid Mediators and Tumor Intracrinology

**DOI:** 10.3389/fendo.2018.00594

**Published:** 2018-10-26

**Authors:** Rachel Bleach, Marie McIlroy

**Affiliations:** Endocrine Oncology Research Group, Department of Surgery, Royal College of Surgeons in Ireland, Dublin, Ireland

**Keywords:** androgen receptor, breast cancer, steroids, intracrinology, non-genomic signaling

## Abstract

Androgen receptor (AR) is the most widely expressed steroid receptor protein in normal breast tissue and is detectable in approximately 90% of primary breast cancers and 75% of metastatic lesions. However, the role of AR in breast cancer development and progression is mired in controversy with evidence suggesting it can either inhibit or promote breast tumorigenesis. Studies have shown it to antagonize estrogen receptor alpha (ERα) DNA binding, thereby preventing pro-proliferative gene transcription; whilst others have demonstrated AR to take on the mantle of a pseudo ERα particularly in the setting of triple negative breast cancer. Evidence for a potentiating role of AR in the development of endocrine resistant breast cancer has also been mounting with reports associating high AR expression with poor response to endocrine treatment. The resurgence of interest into the function of AR in breast cancer has resulted in various emergent clinical trials evaluating anti-AR therapy and selective androgen receptor modulators in the treatment of advanced breast cancer. Trials have reported varied response rates dependent upon subtype with overall clinical benefit rates of ~19–29% for anti-androgen monotherapy, suggesting that with enhanced patient stratification AR could prove efficacious as a breast cancer therapy. Androgens and AR have been reported to facilitate tumor stemness in some cancers; a process which may be mediated through genomic or non-genomic actions of the AR, with the latter mechanism being relatively unexplored in breast cancer. Steroidogenic ligands of the AR are produced in females by the gonads and as sex-steroid precursors secreted from the adrenal glands. These androgens provide an abundant reservoir from which all estrogens are subsequently synthesized and their levels are undiminished in the event of standard hormonal therapeutic intervention in breast cancer. Steroid levels are known to be altered by lifestyle factors such as diet and exercise; understanding their potential role in dictating the function of AR in breast cancer development could therefore have wide-ranging effects in prevention and treatment of this disease. This review will outline the endogenous biochemical drivers of both genomic and non-genomic AR activation and how these may be modulated by current hormonal therapies.

## Overview of review

The steroid nuclear receptor superfamily encodes proteins that selectively bind lipid and cholesterol derived ligands. Vertebrate steroid nuclear receptors include the estrogen receptor alpha (ERα), androgen receptor (AR), glucocorticoid receptor (GR), progesterone receptor (PR), and mineralocorticoid receptor (MR) (1). Steroid receptor ligands are highly lipophilic in nature and as a consequence bind to a hydrophobic cavity in the α-helical fold (2), this in turn, controls coregulatory interactions by inducing allosteric changes on the receptor surface. Many studies in hormone driven cancers focus on the expression of nuclear receptors however, arguably it is the level of steroid and relative affinity for the receptor present that is more pertinent as they are the vital stimulus required for receptor activation and cellular responses (3). Many breast cancer studies have shown that serum estrogens and androgens are associated with both increased and decreased breast cancer risk (4–6). Although much research in breast cancer has focused on ERα and its steroid ligands it has been shown that in subsets of ER and PR negative primary breast carcinomas, molecular profiles indicate hormonally regulated transcription still occurs (7). This suggests the involvement of other sex steroids and/or their receptors such as the most abundantly expressed nuclear receptor in breast cancer, AR. Renewed focus on AR in breast cancer has shown that alterations in the antagonist relationship between ERα and AR may play a role in the development of breast cancer, and in particular, skewed AR ERα expression ratio may be a factor that influences the development of resistance to ERα -directed therapy (8–10). Here, we describe recent findings on AR in breast cancer with a renewed interest in elucidating the role of sex steroids and their metabolites in disease progression.

## AR and androgens in normal breast tissue and breast cancer

### AR in normal mammary gland

**Figure 1:**
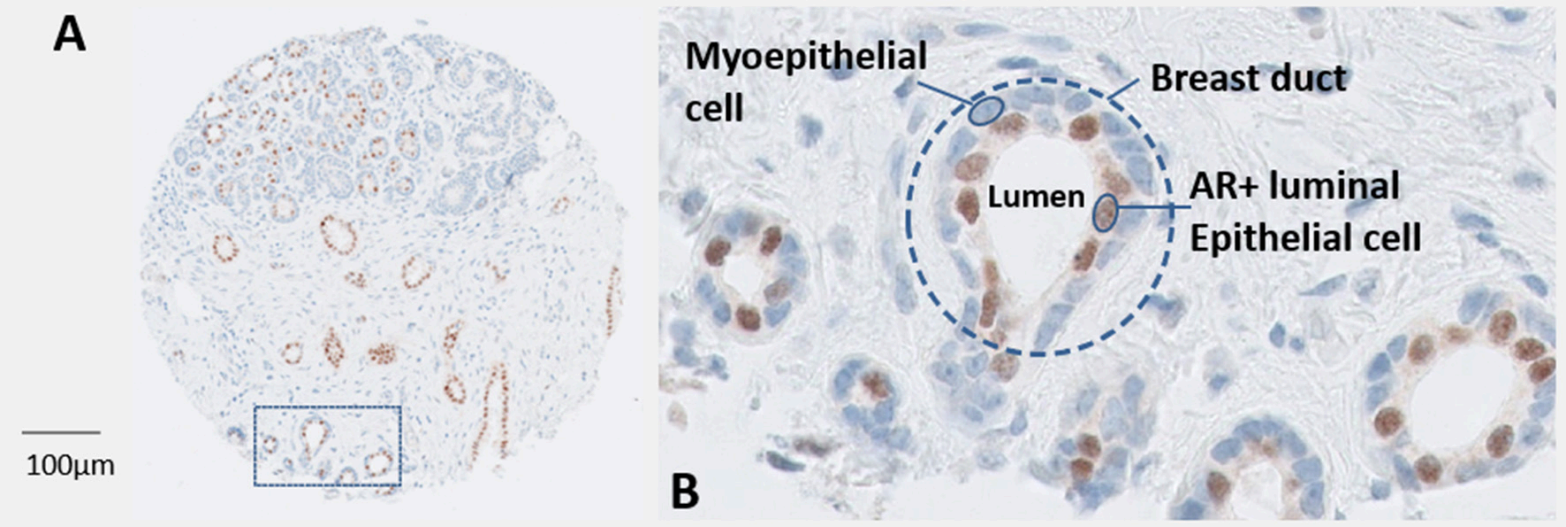
**(A)** A core from a breast tumor tissue microarray depicting normal ductal structures within the mammary gland that have been stained immunohistochemically for AR (Novocastra, Leica). **(B)** A magnified region of normal breast ducts. The outer myoepithelial layer of cells are devoid of AR, in contrast ~30–40% of the inner luminal epithelial cells express high levels of AR protein.

The adult human mammary gland is a dynamic structure consisting of glandular alveoli, grouped to form ~20 lobules that are corralled by adipose and connective tissue and drain into ductal structures that terminate at the nipple. The mammary gland is a target tissue for a variety of hormonal stimulus arising from the hypothalamo-pituitary axis with steroid hormones dictating the concerted cyclical remodeling of the gland over a woman's lifetime from menarche to menopause. The development, maturation, and involution of the mammary gland are under tight regulation by the endocrine system with steroid nuclear receptors expressed in ~30% of the epithelial cells lining the ductal lumen (11). The AR is the most abundant nuclear receptor in mammary epithelial cells with the majority of luminal cells also co-expressing ERα and PR (11) (Figure 1).

### Fate of sex steroid precursors and their metabolites in breast tissue

In women, sex steroids are secreted from the ovaries and from the adrenal cortex. Both these tissues share a common origin during embryogenesis, the coelomic mesothelium, hence the adrenal glands can be referred to as being bi-sexual accessory sex organs due to their capacity to secrete the major sex steroid precursors dehydroepiandrosterone (DHEA) and androstenedione (4-dione) (12, 13). During adrenarche (~age 6–8) the adrenal glands ramp up secretion of DHEA and DHEA-S with levels peaking during the third decade of life before steadily declining to around 5% of their maximum levels in old age [reviewed (14)]. Humans and primates are unique in their capacity to produce large amounts of adrenal steroids. These steroids are then metabolized to sex hormones (estrogens and androgens) in peripheral tissues where they bind to specific receptors and mediate physiological response.

**Figure 2:**
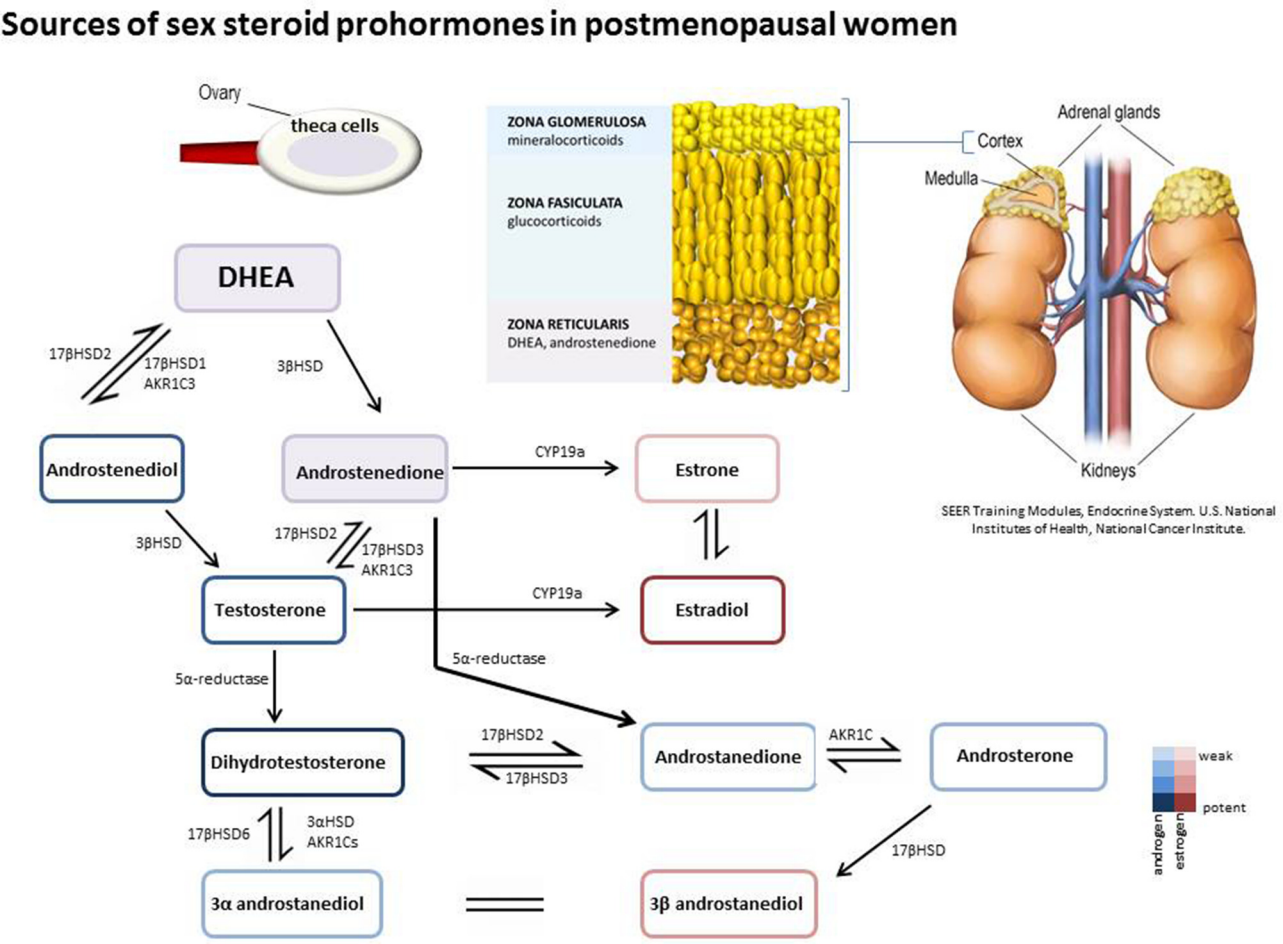
Prohormones are secreted from the adrenal zona reticularis and ovarian theca cells in post-menopausal women. DHEA and 4-dione can be further metabolized intracellularly in peripheral tissues by hydroxysteroid dehydrogenase/isomerases, reductases, and aromatase to generate both androgenic and estrogenic metabolites.

Dehydroepiandrosterone sulfate (DHEAS), DHEA, and 4-dione are the main circulating sex steroid precursors within females and are listed here in order of abundance (15). 4-dione is the direct sex steroid precursor for both androgens and estrogens with reference range levels in females exceeding that of males. 4-dione differs in bioavailability in comparison to the other steroid precursors (DHEA and DHEAS) as levels of 4-dione fluctuate during the menstrual cycle due to significant ovarian secretion (16). The primary sources of 4-dione in women are from the adrenal (50%) and the ovarian stroma (50%) (15); of note, whilst ovarian shut-down results in a dramatic decrease in estrogen production the ovaries retain a degree of their androgen producing capabilities post-menopause (17–19). Circulating adrenal hormones are often transported as fatty acylated ester derivatives within lipoproteins (20, 21). Once within the peripheral tissues they may be stored in their esterified form or may become further metabolized to act as classical steroid receptor ligands and drive genomic steroid signaling (overview Figure 2). Steroid homeostasis within the cell is dictated by the amount of glucoronidation or sulfation which acts as the primary mechanism for de-activation of steroids (22, 23).

### Steroid prohormones and metabolism in breast cancer

There is mounting epidemiological and historical evidence that suggests estrogens may not be the sole steroid drivers of breast cancer (24–27). A major deficiency in our understanding of breast cancer intracrinology is a comprehensive knowledge of sex steroid precursor metabolism. This is particularly pertinent in the context of resistance to aromatase inhibition wherein the tumor microenvironment will become depleted of estrogens. Aromatase inhibitors (AIs) are currently gold-standard first line therapy for the treatment of post-menopausal breast cancers that express ERα. AIs are a very effective class of drugs, which inhibit the action of the aromatase enzyme. Aromatase is responsible for the aromatization of the weak androgen, 4-dione, into estrone (E_1_) which can then be further metabolized into the highly estrogenic steroid, estradiol (E_2_).

Importantly, prohormones such as DHEA and 4-dione can also be metabolized to the androgen testosterone or to androstanedione by reductase in the absence of aromatase. Indeed, studies have highlighted a protumorigenic effect of such derivatives e.g., androstanedione and androstanediol in breast cancer models (28). Another DHEA metabolite, androstenediol, has also been implicated as a driver of tumorigensis in breast cancers with low intra-tumorial estradiol levels (29), highlighting the relevance of individual tumor intracrinology. A recent study by Moon et al. (30) has made great strides in accurately quantifying steroid levels within breast cancer tumors and, of note, whilst levels of prohormones were the most abundant they also reported huge amounts of variation among patients emphasizing this as a key area of future research. It is therefore of great interest to understand how circulating sex-steroid precursors exert their role on breast tumor survival, particularly in the setting of anti-estrogen therapy failure. This is most pertinent when we consider the fact that these compounds are inherently weak androgens with an affinity for the AR which surpasses that of all other steroid receptors that may be present within the tumor.

### Hormone-dependent breast cancer

Since Beatson reported on the utility of oophorectomy in the treatment of advanced breast cancer in 1896 there has been a firm focus on steroid ablation therapy in the treatment of this disease (31). The early twentieth century saw major advances in our understanding of the endocrine system, a term which encapsulates steroid hormone secretion from the gonads, adrenal cortex, thyroid, and pancreas which are under tight adeno-hypophysis regulation. Each of these endocrine organs produce hormones which will then circulate in the blood before reaching their specific target organs. Breast tissue is a target organ for ovarian hormonal stimulus during the premenopausal period, however, after ovarian shut-down hormonal stimulus will arise from the adrenal cortex. It was through the work of Huggins that the importance of the adrenal steroids in the breast and prostate cancer was elucidated. This resulted in the utilization of bi-lateral adrenalectomy in the treatment of these cancers prior to the development of modern endocrine therapeutics (32, 33). With the identification of the protein receptor target (namely ERα) for estradiol there was a significant breakthrough in the identification of a robust biomarker for hormone dependent breast cancer (34, 35). Indeed, throughout the ensuing decades numerous anti-estrogen therapies have been devised and successfully delivered into the clinical milieu where they have saved thousands of lives. Hormone receptor positive tumors account for approximately 75% of all breast cancer diagnoses (36), and since the development of robust clinical antibodies and therapies targeting either the ER protein or the enzyme aromatase (Cyp 19a) responsible for estrogen synthesis, mortality rates for ERα +ve tumors have been successfully attenuated. Unfortunately, despite the huge advances in treatment, over one third of women will eventually suffer recurrence of their disease. The drive to understand mechanisms underlying resistance to these therapies is a major concern due to the large numbers of women affected and the deficit in determining who will exhibit a sustained response to hormonal therapy and who will not. From the past number of decades it is apparent that altered response to hormonal stimulus is a hallmark of these tumors with a shift towards growth factor dependency often a common feature (37–41). Recently, there has been a resurgence of interest in elucidating the role of other nuclear receptors, and indeed other hormones, in driving breast tumorigensis (30, 42, 43). Many studies have centered upon the potential role of AR, however, due to its ubiquitous presence within breast cancers this is proving to be challenging (40). It is therefore of interest to understand the normal role of AR in mammary gland biology, its endogenous ligands and localization.

### AR in breast cancer

**Figure 3:**
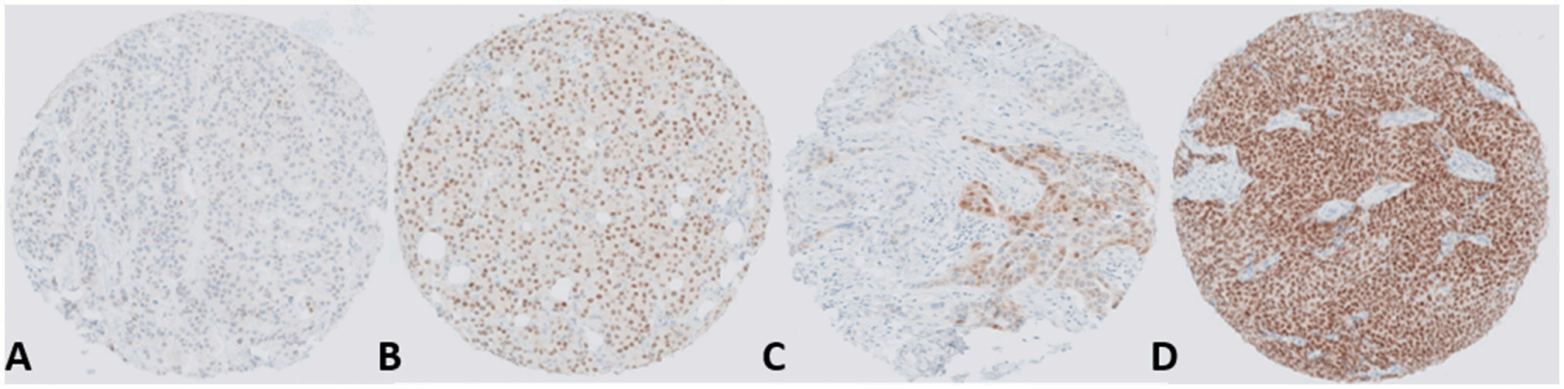
Representative images depicting the range and heterogeneity of AR protein expression within breast tumors. The panel shows ER+ve positive tumors with **(A)** Low AR expression, **(B)** Moderate AR expression, **(C)** Heterogeneous AR expression and **(D)** High AR expression.

Numerous studies have reported that approximately 80% of breast cancers are positive for AR, furthermore AR protein is detectable across luminal A (ERα+ve, PR+ve, Her2-ve), Luminal B (ERα+ve, PR+ve, Her2+ve), triple-negative/basal (ER-ve, PR-ve and Her2-ve) and Her2 amplified (Her2+) (13, 44). The majority of AR positive tumors are ERα positive (~90%); however AR is also detectable in a significant proportion of triple-negative and basal tumors (~35–50%) (10, 45, 46). AR expression has been associated with good patient outcome in a large number of clinical datasets (44, 47). Preclinical studies have surmised that AR may enact its protective role by blocking ERα gene transcription (48), however, other studies in triple-negative and apocrine breast cancers indicate that AR may act as a pseudo- ERα in this setting (49, 50). There has also been some confounding clinical data in which AR positivity indicates lack of complete clinical response to neoadjuvant chemotherapy in contrast to patients who are negative for both AR and ERα (51). In recent years there has been a growing interest in targeting the AR in breast cancers either by utilizing AR agonists such as enobosarm or by antagonizing AR actions using drugs primarily developed as therapeutic agents for prostate cancer which have been repurposed in the past number of years, specifically bicalutamide, enzalutamide and the CYP17 inhibitor, abiraterone. Results from these trials have been varied with better outcomes reported in AR positive triple negative tumors (LAR—luminal AR subtype of triple negative breast cancer). Nevertheless, in hormone receptor positive advanced breast cancers there is a significant proportion of patients who respond favorably to anti-AR therapy. A major hurdle in successful utilization of these drugs in the treatment of breast cancer is our lack of understanding at what drives this dichotomy—what makes some AR positive tumors behave so differently and respond in such a different way to the same treatment? Immunohistochemical studies of breast cancers have shown that whilst the majority of tumors express some degree of AR protein (>1%) there is also a huge diversity in the percentage of cells within a tumor expressing the protein and also the relative abundance of the protein present (Figure 3). A recent meta-analysis by Ricciardelli et al. (44) highlighted the importance of evaluating the abundance of the AR when determining its impact on breast cancer outcome.

### Targeting AR in the treatment of breast cancer

Currently there are ~16 active/recruiting clinical trials of drugs targeting the AR in the treatment of breast cancer across various subtypes (Table 1). The majority of these compounds are repurposed AR antagonists presently utilized in the treatment of prostate cancer.

**Table 1:** A compendium of clinical trials either currently active or recruiting which target AR in breast cancer.

**Therapeutic agent**	**Clinical trial**	**Status**	**Breast cancer subtype**	**Eligibility criteria**	**Clinical trial phase**
Pembrolizumab and Enobosarm^*^	NCT02971761	Recruiting	Metastatic triple negative breast cancer	AR+ve (AR expression ≥10% nuclear AR). ER−ve (ER expression ≤ 1% positive tumor nuclei), PR−ve (PR expression ≤ 1% nuclear PR) & HER2−ve	Phase 2
EMERALD trial-Enobosarm^*^ (Windows Study)	CRUK/15/075	Recruiting	Early stage breast cancer	Postmenopausal AR+ve (≥10% nuclear AR staining), ER+ve (Allred ≥3), Any HER2 status	Phase 2
RAD140^*^	NCT03088527	Recruiting	Locally advanced or metastatic breast cancer.	Postmenopausal hormone receptor positive, HER2 negative	Phase 1
CR1447 (4-0H-testosterone)^*^	NCT02067741	Active	Endocrine Responsive-HER2 Negative and triple negative Metastatic or locally advanced Breast cancer	Postmenopausal Stratum A: endocrine responsive: HER1−ve, ER+ve ≥1%, PR+ve ≥1%, HER2−ve or ER+ve ≥1%, PR−ve <1%, HER2−ve. Stratum B: triple negative: ER −ve <1%, PR−ve <1%, HER2−ve and AR+ve >0%	Phase 2
Orteronel^#^	NCT01990209	Recruiting	Metastatic breast cancer	Category 1: triple negative: ER−ve, PR−ve, HER2−ve. Category 2: Pre-menopausal with ovarian suppression or post-menopausal: ER+ve, PR+ve, and HER2+ve. All AR+ve ≥10%.	Phase 2
Seviteronel^#^	NCT02580448	Recruiting	Advanced breast cancer	ER+ve ≥1% and HER2 normal, or triple negative breast cancer (ER−ve/PR−ve- if 0% by IHC and HER2 normal)	Phase 1/2
Darolutamide^∧^ -START	NCT03383679	Recruiting	Triple negative locally recurrent or metastatic breast cancer	ER−ve and PR−ve ≤ 10% tumor, HER2−ve, AR+ve * =* ≥10% tumor stained cells	Phase 2
BVL719 (Aipelisib) and Enzalutamide ^∧^	NCT03207529	Not yet recruiting	Metastatic breast cancer	ER and/or PR+ve, HER2−ve or ER−ve, PR−ve, HER-2 negative. AR-positive ≥1% of nuclear staining and PTEN+ve >0% of nuclear staining	Phase 1
Bicalutamide^∧^ plus AI	NCT02910050	Recruiting	Metastatic breast cancer	Postmenopausal ER+ve, AR+ve and HER2−ve	Phase 2
Enzalutamide^∧^ plus Taxol	NCT02689427	Recruiting	Triple negative breast cancer	ER−ve ≤ 10%; PR negative ≤ 10% and HER2 0-1 +(FISH non amplified) AR+ve ≥10% of nuclear staining	Phase 2
Taselisib and Enzalutamide ^∧^	NCT02457910	Active	Triple negative metastatic breast cancer	Phase lb: HER2−ve, ER/PR −ve/+ve. Phase II: ER−ve <1%, PR−ve <1%, HER2−ve, AR+ve ≥10% of tumor nuclei	Phase 1b/2
ODM-201^∧^ (Presurgical Study)	NCT03004534	Recruiting	Invasive breast cancer	Known ER, PR, and HER2 statuses.	Early phase 2
Bicalutamide^∧^	NCT03055312	Recruiting	Metastatic triple negative breast cancer	Triple negative breast cancer, AR positive ≥10% tumor cells	Phase 3
Bicalutamide^∧^	NCT00468715	Active	ER, PR negative metastatic breast cancer	ER and PR−ve ≤ 10% of tumor cell nuclei. AR+ve ≥10% of tumor cell nuclei	Phase 2
Nivolumab, lpilimumab and Bicalutamide	NCT03650894	Not yet recruiting	Advanced breast cancer	HER2-negative breast cancer	Phase 2
Enzalutamide^∧^ alone or in combination with exemestane (Windows study)	NCT02676986	Recruiting	Patients with primary breast cancer	PostmenopausaiER+ve cohort: ER+ve ≥1% of tumor cells positive. Triple negative cohort: AR+ tumors− any nuclear AR staining, ER−ve <1% of cells, PR−ve <1% of tumor cells, HER2 with 0, 1+ or 2+ intensity on IHC and no evidence of amplification of the HER2 gene	Phase 2
Palbocidib with Bicalutamide	NCT02605486	Recruiting	Metastatic breast cancer	ER/PR+ve ≥1% or ER/PR−ve <1%, HER2 normal. AR+ve ≥1%of cell nuclei	Phase 1/2
Ribociclib & Bicalutamide^∧^	NCT03090165	Recruiting	Advanced triple negative breast cancer	Triple negative breast cancer with AR positivity >0% staining of tumor nuclei	Phase 1/2
Enzalutamide^∧^	NCT02750358	Recruiting	Early stage triple negative breast cancer	Triple negative breast cancer: ER negative <1%, PR−ve <1% and HER2 0 or 1 +or FISH not amplified if IHC2+.AR+ve ≥1 % nuclear staining	Phase 2

* Androgen receptor agonists- SARMS.

# Androgen synthesis inhibitors.

∧ *Androgen receptor antagonists*.

It may appear unusual that AR agonists, antagonists and androgen synthesis inhibitors are all under development for the treatment of breast cancer. However, we must consider that prior to the introduction of anti-estrogen therapies, both estrogens and androgens were frequently administered at supraphysiological concentrations as treatment for breast cancer. In recent times, the use of steroids has fallen into disfavor in the clinic. This can probably be attributed to the introduction of tamoxifen in the late 1970's, followed more recently by AI therapies, but primarily due to the unfavorable side effect profile associated with steroid treatments. As AR has a dichotomous role in breast cancer, AR agonists, or antagonists may offer therapeutic benefit perhaps dictated, in part, by subtype specificity. Selective Androgen Receptor Modulators (SARMs) such as enobosarm are being explored for clinical use as they may counteract the activity of ER in driving breast cancer growth (Table 1). Enobosarm is more likely to be effective in hormone receptor positive breast cancers as a trial analyzing its utilization in triple negative breast cancer was terminated due to lack of efficacy (ClinicalTrials.gov Identifier: NCT02368691). CR1447 (4-hydroxytestosterone, 4-OHT; NCT02067741) is an ointment that is being assessed in the treatment of ER+ve, AR+ve and HER2-ve advanced breast cancer. It works in two ways, as a steroidal aromatase inhibitor, and has a high binding affinity for AR. In a phase 1 trial it was well tolerated when administered transdermally as an ointment, and also displayed single agent activity in heavily pretreated ER+ve/HER2-ve breast cancer patients (52).

In general, the majority of clinical trials are exploring AR antagonists as a therapeutic option for breast cancer. Enzalutamide (Xtandi) is a compound that inhibits AR signaling through a number of different mechanisms of action. It exerts its effect by inhibiting the binding of androgen ligands to AR, by inhibiting nuclear translocation of AR and also by preventing binding of AR with DNA. Clinical trial data was presented on the first randomized trial of enzalutamide at San Antonio Breast Cancer Symposium 2017 (53). The results showed a significant increase in progression free survival (PFS) of 16.5 months for patients with hormone receptor positive advanced breast cancer who had received no prior endocrine therapy when treated with a combination of exemestane and enzalutamide. This was compared to 4.3 months PFS in the control arm. The patients were identified based on a gene based signature biomarker known as Dx, which was used to indicate AR signaling. Interestingly, significant improvement was not noted in the cohort with one prior endocrine therapy (54). Enzalutamide is also being evaluated for the treatment of triple negative breast cancer. A phase 2 study assessing the clinical activity and safety of enzalutamide in patients with advanced triple negative breast cancer found that it is well tolerated and clinically active (ClinicalTrials.gov Identifier: NCT01889238). The authors report that >0% AR staining was detected in 80% of tumors and ≥10% AR nuclear staining was detected in 55% of tumors. In this study >0% AR staining was considered positive. The intent-to-treat (ITT) population included all enrolled patients. It is interesting to note that in this group, a clinical benefit rate (CBR) of 25% and median overall survival of 12.7 months was observed. However, in the evaluable subgroup, which included patients with ≥10% AR, a CBR of 33% and 17.6 months median overall survival was reported (55). This highlights one of the pressing issues with clinical trials evaluating AR targeted therapies in breast cancer. There is a lack of clarity when deciding a cut-point for AR positivity. This enzalutamide clinical trial suggests the likelihood of patients achieving clinical benefit to anti-AR therapies may be directly related to the level of AR expression within the tumors. Recently released data from a phase 2 clinical trial assessing the efficacy and safety of enzalutamide with trastuzumab in patients with HER2+ve, AR+ve metastatic breast cancer (ClinicalTrials.gov Identifier: NCT02091960) looks promising. The study is ongoing but results released earlier this year showed a clinical benefit rate of 23.6% and progression free survival of 105 days. Another AR antagonist bicalutamide was under investigation in a phase II clinical trial of triple negative breast cancer. AR positivity as defined as >10%. In this study a 19% clinical benefited rate was observed (56).

Abiraterone acetate is an irreversible inhibitor of the enzyme CYP17A1 (17α-hydroxylase C17,20-lyase). Abrogating the effects of CYP17 depletes the synthesis of both estrogens and androgens. Abiraterone acetate was assessed in a cohort of 293 metastatic ER+ve patients. The trial was designed so that patients would receive either abiraterone plus prednisone and exemestane, abiraterone plus prednisone or exemestane on its own. This study did not find any improvement in PFS compared to exemestane alone. The authors suggest that the increase in progesterone as a result of abiraterone acetate mechanism of action may have contributed to this negative result (57). Furthermore, a complete lack of steroids may not be therapeutically beneficial in breast cancer. A review of the literature by Alferez et al. suggests a lack of estrogen and progesterone hormones may increase cancer stem cells and resistance to therapy (58). Abiraterone acetate plus prednisone was also assessed in a small phase II clinical trial of patients with metastatic or locally advanced triple negative breast cancer. This trial did not meet its target CBR at 6 months. However, they did report that benefit of the treatment could be noted in some patients with molecular apocrine like tumors (59).

Overall, anti-androgen therapies have displayed a relatively modest CBR in clinical trials. A major concern with clinical trials assessing the efficacy of anti-AR therapies in breast cancer is that, currently, we do not have a standardized cut-point for AR positivity. As highlighted in Table 1, eligibility criteria for clinical trials ranges from AR positivity at 1% to >10% with the use of various different antibodies and detection methods. Furthermore, even the assessment of levels of nuclear receptors (ER and PR) used in breast cancer subtyping is not consistent between studies.

There are a number of recruiting and ongoing clinical trials targeting AR in breast cancer as outlined in Table 1. Anti-AR therapy for the treatment of breast cancer is a very exciting field and we wait in hopeful anticipation for the emergence of more clinical trial data. However, currently the major limitations of these studies appear to be, the lack of consistency in determining a level of positivity for hormone receptors, exclusive calling of nuclear staining and the absence of a robust clinical biomarker to better direct the use of AR directed therapies.

## Genomic AR signaling

### AR co-regulatory proteins

Nuclear receptors cannot control transcriptional activation on their own, they require co-activators and co-repressors to do so. Therefore, co-regulators are implicated in a diverse number of cellular functions. With hundreds of nuclear receptor co-regulators identified it is beyond the scope of this manuscript to detail all the AR co-regulators however some of which have been implicated in breast cancer are discussed in detail below.

Many AR co-regulatory proteins have been identified in prostate cancer and there is substantial overlap with those identified in breast cancer, however not necessarily associated with AR as of yet. Magklara et al. conducted research on a panel of breast cancer cell lines and showed that although PSA and human glandular kallikrein (KLK2) are androgen regulated genes, differential expression was not related to levels of AR expression. However, nuclear receptor co-regulators displayed distinct patterns of expression suggesting co-regulator expression can significantly influence AR target gene expression (60).

The first AR co-activator identified was Androgen Receptor Associated protein 70 (ARA70) in prostate cancer (61). ARA 70 works by increasing AR expression, protein stability and nuclear translocation. ARA 70 has also been shown to interact with ERα and may play a role in modulating AR and ERα activity in MCF7 breast cancer cells (62). It is recruited to known ERα target genes and enhances ERα transcriptional activity. Interestingly, the authors of this paper found that the inhibitory effects of AR on MCF7 cells can be overruled by overexpression of ARA70. Furthermore, coimmunoprecipitation experiments found that higher levels of ERα to AR (Ratio 5:1) lead to ARA70 coimmunoprecipitating with ERα, however when the ratio is reversed, ERα to AR 1:5 ARA70 coimmunoprecipitates with AR (62).

The most widely studied steroid receptor coactivator family in breast cancer are the p160 kDa group of SRC −1 (NCOA1), −2(NCOA2), and −3 (NCOA3/AIB1). These interact with the ligand binding domain of nuclear receptors through the LXXLL motif. SRC1 and SRC3 have been shown to be overexpressed in a number of breast cancer studies. SRC1 promotes cell growth and tumor progression in prostate cancer (63) and it has also been implicated in endocrine treatment resistance in breast cancer (64, 65). In prostate specific-antigen (PSA) expressing breast cancer cells, SRC1 mRNA levels correlate with PSA secretion (60). SRC-3 (AIB1) is a known AR co-activator in prostate cancer (66), and is associated with a poor prognosis in breast cancer (67). Park et al. demonstrated that breast cancer type 1 susceptibility protein (BRCA1) is a coactivator of the AR and together with the P160 co-activators may modulate AR signaling through direct interaction with the AF1 domain in both breast and prostate cancer. Cotransfection of BRCA1 with p160 coactivators enhanced AR signaling (68). Androgens have also been shown to be able to regulate the expression of AR coregulators in prostate cancer. These include SRC-3, CBP, Male Germ Cell-Associated Kinase (MAK), BRCA1 and β-catenin which may enhance AR signaling though feedback mechanisms (69). Many of these genes have been implicated in breast cancer and coupled with the dichotomous levels of androgenic steroid present, this warrants further investigation particularly in the context of active tumor promoting AR signaling.

There are many coregulator and AR protein interactors that have been identified in prostate cancer which are also expressed and implicated in many functions in breast cancer however there is little experimental evidence on the role of these proteins in breast cancer subtypes. A comprehensive list of androgen receptors interacting proteins and co-regulators identified before 2010 can be found at http://androgendb.mcgill.ca/ARinteract.pdf. Although breast and prostate cancer are similar in that they are both hormonal driven diseases, it is important to elucidate the role of coregulator and interacting protein in each type of cancer and additionally in different patient subsets.

### AR cistrome

The importance of understanding steroid nuclear receptor binding profiles is becoming more and more recognized as it has been shown to indicate patient outcome in both breast and prostate cancer (70, 71). In 1988, Ham et al. reported on a 15 base pair oligonucleotide sequence identified as an androgen response element (ARE) but it also acted as a response element for PR and GR (72). Subsequent studies showed that all class 1 steroid receptors, AR, GR, PR and MR bind to a consensus response element which is composed of inverted 6 base pair repeats separated by two nucleotides 5′ AGAACA nnn TGTTCT 3′ (73). Although AR, GR, PR, and MR can all recognize and bind to the same consensus sequence, specific binding sites which diverge from this also exist for each receptor (74). These distinct sites permit specificity of receptors for target genes. Claessens et al. was one of the first to recognize AR specificity for an ARE and demonstrate responsiveness to androgens (75). Subsequently, it was demonstrated that AREs which are more explicit for AR than GR, consist of a non-conventional ARE with imperfect direct repeats of two core binding elements (76). In addition to this, it has been shown that specific structural interactions with the response element (77), transcription factors present (78) and the ability to interact with direct repeats of 5′ TGTTCT 3′ (73) also dictate exclusive AR binding. However, this is not the complete picture of AR binding as it is far more complex. Chromatin immunoprecipitation (ChIP) sequencing data in prostate cancer cell lines has provided evidence that the majority of AREs contain several differing base pairs to that of the reported palindromic ARE full site (79). Furthermore, ARE binding can be influenced by cooperating factors and adjacent motifs, particularly in the case of ARE half sites (79).

There is still much debate surrounding nuclear receptor transcription mechanisms and some contrasting results between AR chromatin binding in breast cancer cell lines vs. human tissues have recently been published (80). This study analyzed the interplay between steroid receptors and pioneer factors in male breast cancer. They reported that there is a stronger correlation between ER and AR clustering in individual tumors compared to inter-tumorally, suggesting subtype specificity and more importantly patient specificity (80). AREs and adjacent transcription factor binding sites have been extensively studied in prostate cancer however, little is known about their mechanisms in breast cancer particularly *in vivo*. For this reason and other contributing factors, the clinicalpathological importance of AR in breast cancer has been marred with conflicting results.

### Ligand regulation of AR:DNA interaction

There is a dynamic, context dependent relationship between AR and ER DNA binding in breast cancer. Like AR, ER binds to 3 nucleotide palindromes, with the recognition sequence 5′ TGACCT 3′ (81). Although AR has a different consensus response element, electrophoretic mobility shift assays and ChIP studies have found AR to be able to compete with ER for binding to the estrogen response element (ERE) (48).

**Figure 4:**
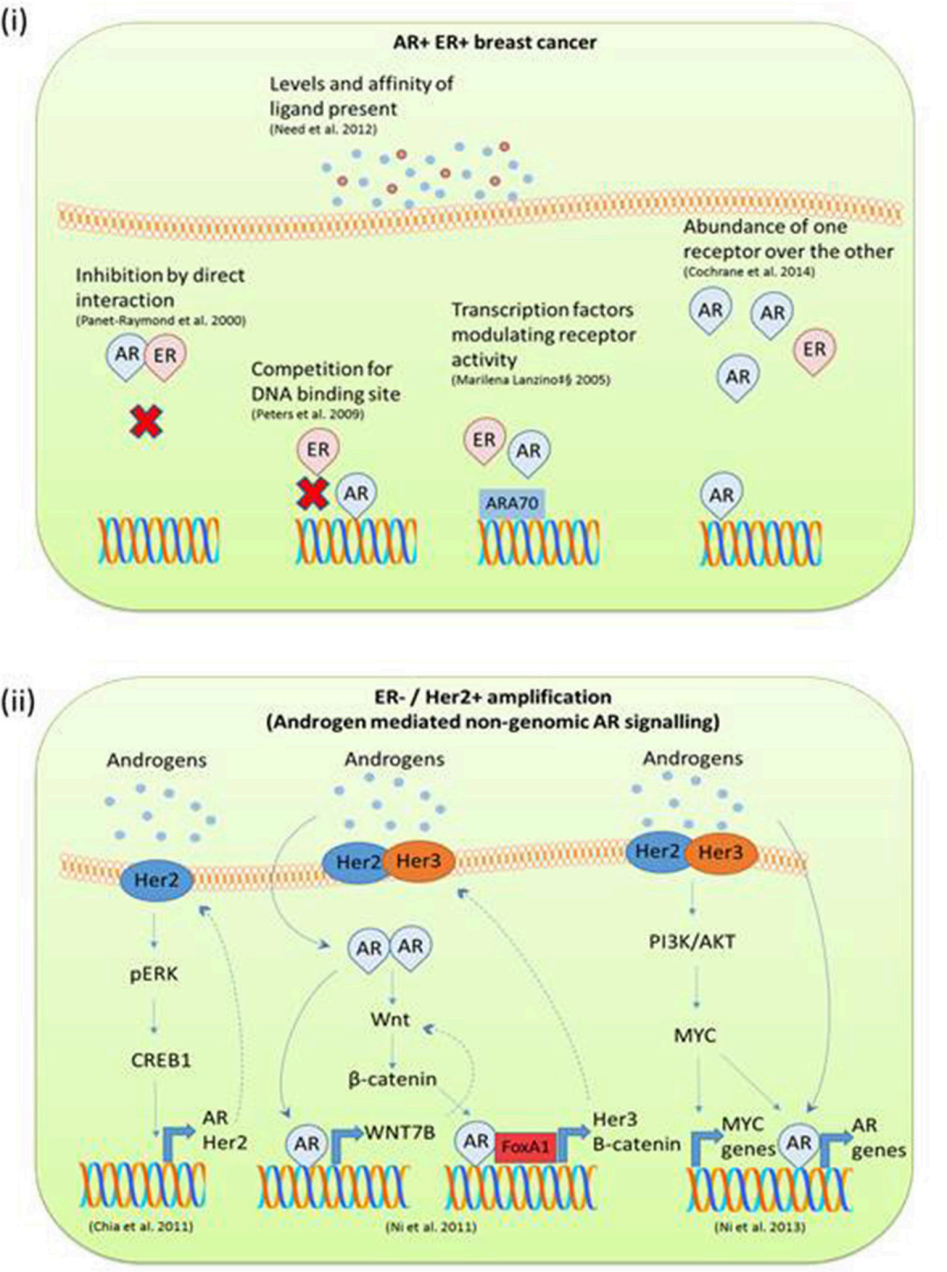
(i) Mechanisms of AR ERα mediated gene transcription in AR+ve ERα +ve breast cancer. Many studies have reported on the dynamic relationship between ER and AR DNA binding and direct interactions in breast cancer. AR and ERα can transcriptionally regulate each other through heterodimerization and binding to the same DNA sequence. AR ER target gene transcription is also influenced by the level of steroid present and overexpression of receptors. (ii) Mechanisms of androgen mediated signaling reported in ERα negative Her2 amplified breast cancer studies. In triple negative breast cancer with Her2 amplification, androgens appear to initiate 2^nd^ messenger signaling cascades. This often results in a feedback loop and in this way drives tumor progression.

It has been widely documented that androgens can inhibit mammary cell proliferation, whereas estrogens stimulate growth of breast cells (36, 82). Clinically AR protein expression has been associated with a good prognosis in early stage breast cancer (83). Also in luminal A (ER+ve, AR+ve) breast cancer MCF7 cells, the cyclin D1 promoter has been identified as harboring a functional ARE. DHT stimulation prevents cyclin D1 expression and therefore abrogates its mitogenic effects (84). Indeed there are several reported mechanisms by which estrogen and androgen steroid receptors may antagonize each other and regulate cell growth. Documented examples include: binding to shared response elements on the DNA (48), competition for transcriptional coregulators (62), homo or hetrodimerization (85), and the ability of activating ligands to bind to more than one receptor (3) as depicted in Figure 4 (i). In a seminal study AR was shown to prevent estradiol stimulated ER target gene transcription and growth by binding to a subset of EREs (48). Conversely, AR has been shown to drive breast cancer cell growth in molecular apocrine tumors (triple negative, AR+ve) (86, 87). Indeed, although molecular apocrine tumors have no ER expression, a number of ER target genes are expressed, suggesting that AR can take on the role of a pseudo ER (86). Supporting data from Robinson et al. found that AR can bind ER cis-regulatory elements in the molecular apocrine subset of breast cancer tumors (49). Need et al. has also found that in a luminal breast cancer cell line, AR binding sites that were void of AREs were enriched for the retinoic acid receptor related orphan receptor α motif which also harbored an ERE half site core sequence (88). This demonstrates ARs ability to directly target ERE half sites either via direct binding or by indirect receptor interactions.

Just as EREs are enriched in AR binding sites, AREs have been found to be enriched in ERα binding sites (88). In both male and female breast cancers, the majority of AR sites are also occupied by ERα (80). When cell lines are treated with estradiol, AR binding is enriched for EREs and overlaps with ER binding sites (89). Furthermore, AR has been shown to be required for maximum ER genomic binding as the anti-AR drug enzalutamide decreases ER binding sites by around 50% (89). The dynamics between AR and ER in breast cancer are not just specific to DNA binding. Panet-Raymond et al. demonstrated through yeast and mammalian two hybrid systems that AR and ER can heterodimerise by direct interaction between the C-terminal of ERα and the N-terminal of AR. Through this mechanism they found that both ERα and AR could decrease the transactivation of each other (85).

### Factors impacting the AR:ER dynamic

ER or AR binding can also be influenced by the level of steroid present thereby varying transcriptional responses. In ZR75.1 luminal breast cancer cells ChIP sequencing and gene microarray experiments showed that higher concentrations of DHT preferentially drive AR binding, conversely higher levels of estradiol favor ER binding. In this case, the relative binding of AR and ER to ARE or ERE regulates the summative proliferative and anti-proliferative effects (88). By skewing hormonal homeostasis, one steroid receptor can dominate over the other; a mechanism proposed as an adaptive response to long term AI treatment (40). MCF7 breast cancer cells overexpressing AR have shown increased resistance to AI therapy compared to non-overexpressing cells (90). Clinically, comparison of primary vs. recurrent AI treated breast cancer samples by immunohistochemistry showed increased AR expression with concomitant decrease in ER and PR expression (40). Interestingly, in 434 patients with recurrent breast cancer, higher levels of the AR target gene, PSA, have been associated with poor response to tamoxifen therapy and decreased overall survival (91). This was independent of other clinical factors such as ER and PR status and although not investigated in the study may imply mechanisms of androgen signaling. Work from our lab has found that in AI resistant breast cancer, 4-dione enhances AR recruitment to the promoter of ER target genes (92). This co-operative AR ER role coincides with the findings from the Fuqua lab where they report in AR overexpressing ERα positive cells which are AI resistant, AR and ERα inhibition is needed in order to regain clinical response (90).

In ERα-ve breast cancer Her2 overexpression impacts AR signaling, particularly in molecular apocrine breast cancer (86). Studies exploring the interplay between Her2 and AR suggest the involvement of second messenger signaling cascades. An *in vitro* study of triple negative breast cancer utilizing ChIP and next generation sequencing found that although there is some overlap with the AR cistrome in a prostate cancer cell line and ERα +ve MCF7 cells, a significant proportion of the AR binding sites are specific to cells which are ERα –ve Her2+ve (93). Androgens have been shown to stimulate growth in ERα -ve Her2+ve breast cancer by a number of mechanisms such as upregulation of Wnt and Her2 signaling (93), via an ERK AR mediated feedback loop (94) or through co-operative regulation with MYC (95) as depicted in Figure 4 (ii). This area of research is showing promise as it has recently been reported in both *in vitro* and xenograft models that anti-AR therapy inhibits the growth of Her2+ve breast cancer to an equivalent level as the anti-Her2+ve therapy trastuzumab and therefore could be used as a second line therapy in this setting (96).

## AR localization, non-genomic signaling and functional consequences

### Factors associated with non-genomic AR signaling

Studies on the genomic actions of sex steroid receptors have failed to account for all actions of sex steroid in breast cancer and of particular note, on the mechanisms of treatment resistance to ER antagonists. It is well known that steroids can induce both chronic and acute actions within cells and a wealth of evidence suggests that sex steroids are involved in rapid non-genomic actions which play roles in cell growth and proliferation. Evidence of estradiol inducing rapid non-genomic signaling has been around since the late 1960's where it was observed to rapidly upregulate cAMP in rat uterus (97). In 1998 the first international meeting on rapid responses to steroids hormones was held in Mannheim, Germany. This was the origin of the term Mannheim criteria which could be used to classify rapid non genomic steroid responses (98). However, the non-genomic role of sex steroids in breast cancer has been a relatively unexplored area and a lot of the information we know regarding non-genomic steroid actions has stemmed from research in other fields, in particular neuroscience (99, 100). Pioneering studies in the 1990's carried out by Migliaccio et al. in which they showed estradiol inducing rapid responses through MAP-Kinase and c-Src pathway in MCF7 breast cancer cells have provided insight into the field of non-genomic actions of sex steroids in breast cancer (101, 102). In 1999 Castoria et al. presented evidence of functional effects of non-transcriptional actions of both estradiol and progestin in breast cancer cell mitogenesis (103). More recently, research in field of prostate cancer has given credence to mechanisms of rapid non-genomic androgen action (104). As this area of research has progressed, features, and hallmarks of non-genomic sex steroid signaling have been defined, as described in Table 2.

**Table 2:** Adapted from the review Non-genomic actions of sex steroid hormones by Simoncini and Genazzani (105).

**Criteria for non-genomic steroid actions**	**Examples**
Very Rapid	Non-genomic steroid signaling occurs within seconds to minutes. Example: In MDA-MB-453 cells treatment with DHT results in induction of p-ERK within 10 min (94).
Does not require RNA/Protein synthesis mediated by steroid receptor	The effects precede and do not require receptor nuclear translocation and RNA or protein synthesis. Example: Effects are seen where canonical gene transcription does not occur (106) and with inhibitors of RNA and protein synthesis such as with the use of Actinomycin D or alpha amanitin (107).
May be induced by membrane bound proteins	Steroids do not have to cross the cell membrane to induce effects Example: If steroids are conjugated to large molecules such as BSA they cannot cross the cell membrane but still bring about effects (108, 109).
Presence of classical steroid receptor is not required	Effects are observed in cells that do not possess classical steroid nuclear receptors Rapid and transient increases in [Ca2+]i have been reported in cells absent of AR expression (110).
Occurs in cells with little or no transcription or translation mechanisms	Can occur in cells with highly compacted chromatin, in which RNA and protein synthesis mechanisms are absent Example: Steroid responses have been detected in sperm cells in which transcription does not occur and platelets which are anucleate (111).
Mutations in the DNA binding domain	Steroids can induce effects even if their classical nuclear receptor has a mutation inhibiting it DNA binding or initiating gene transcription Example: *in vivo* mouse experiments demonstrated androgen induced rapid phosphorylation of ERK1/2 in presence of mutant AR lacking the 2^nd^ zinc finger of the DBD (112).
Cooperation between genomic and non- genomic actions	Studies have shown non genomic actions of nuclear receptors can act in concert or can directly influence genomic nuclear receptor actions or may occur sequentially. Example: The feedback loop between AR expression and Phosphorylation of ERK1/2 (94).

### Ligand activation of non-genomic AR signaling

There is increasing evidence to suggest that androgen ligands can mediate extranuclear pathways, which may be AR dependent and AR independent transcriptional signals. The majority of studies have reported on androgen induced rapid intracellular calcium [Ca^2+^]i changes in a range of cell models (113–115). A recent study in triple negative breast cancer has identified the Ca^2+^ activated K^+^ channel—K_ca_1.1 as an androgen target gene. K_ca_1.1 is associated with breast cancer invasion and metastasis and treatment with anti-androgens prevents its activity (116). Studies in prostate cancer have reported on many mechanisms of rapid non genomic 2nd messenger signaling following exposure to androgens (117, 118). Furthermore, non-genomic AR signaling has been reported in the sertoli cells of the testes (119), fibroblasts (106), osteoblasts and osteocytes (120), stromal cells (121), and breast cancer cells. ERα and AR have been shown to directly interact with the SH2 and SH3 domain respectively of Src. It was found that both DHT and synthetic testosterone (R1881) stimulation of LNCaP prostate cancer cells and MCF7 breast cancer cells results in AR interaction with C-Src (122). The non-genomic role of AR and Src is further supported by studies in a human fibrosarcoma cell line which expresses AR. These cells do not exhibit AR to ARE binding or AR nuclear translocation yet targeting AR decreases tumor migration and proliferation (123). AR has also been reported to activate MAP kinase and this response is insensitive to anti-androgen therapy (117). In a study published by Liao et al. ERK 1/2 phosphorylation was detected within 2 min following DHT treatment. They also found that activated ERK translocates to the nucleus and activates transcription factors, namely ELK1 (124). Androgens have been show to initiate cell motility and invasion in T47D breast cancer cells through proposed non-genomic actions. Phosphorylation of the protein moesin was induced through both ERα and AR, however pertussis toxin inhibited it, suggesting that GPCR plays an initial role (125). In molecular apocrine breast cancer, ERK and AR have a feedback loop that results in co-regulation. Treatment with DHT causes an increase in phosphorylation of ERK1/2 which is dependent on AR expression, conversely, inhibition of ERK1/2 phosphorylation reduces the expression of AR (94). A representative biological model of non-transcriptional AR activity is xenopus laevis oocyte maturation. Androgens are known to be key mediators of this process and effects can occur independently of transcription. Of interest, they found that in this model certain AR ligands, namely 4-dione and testosterone, preferentially induced non-genomic actions however R1881 did not promote non-genomic actions and prevented testosterone effects in the oocytes (126). This suggests an intriguing possibility that specific ligands can instigate differential genomic or non-genomic effects, and this could also be cell type dependent (127).

### Cytoplasmic AR phosphorylation

The AR can be phosphorylated at many sites which can indicate ligand dependent or independent activation and alter AR activity (128). Moreover, AR that has undergone this post translational modification can be located in the cytoplasm or nucleus of cells. AR protein localization in prostate cancer has been found to influence prognosis. It has been reported that levels of phosphorylated AR in the cytoplasm are a stronger prognostic factor than nuclear expression (129). In addition to this, a study exploring the role of phosphorylated AR in breast cancer progression noted that particularly in ERα –ve and invasive ductal carcinoma types, there was higher levels of phosphorylated AR in the cytoplasm than the nucleus (130). This study highlighted the differential expression of phosphorylation sites of AR and in particular its intracellular location, which may influence prognosis. At this current time, we do not have a comprehensive understanding on the specificity of ligands which preferentially induce non-genomic or genomic actions in breast cancer cells. However, a small number of studies have highlighted the differential expression of phosphorylation sites of AR and in particular its intracellular location, which may influence patient prognosis.

Ren et al. found differences in phosphorylation status of AR at serine 213 and serine 650 between benign and malignant breast cancer tissue. They reported increased serine-213 phosphorylation in the nucleus and cytoplasm of breast carcinomas compared to benign breast tissue. This trend was also observed in metastatic breast cancer and invasive ductal carcinomas (130). Nuclear AR-serine(p)-650 was found to be decreased in triple negative breast cancer however cytoplasmic AR phosphorylation at serine 650 was increased 1.4 fold (130). ERK1/2 is known to phosphorylate AR on serine-515 in breast cancer. Phosphorylation at serine-515 with p-ERK1/2 in ERα+ve patients is associated with improved survival. However, when stratified into ERα-ve triple negative breast cancer subtype serine-515 expression correlated with poor prognosis (131). The authors further found that this may be due to an impact on inflammation as ERα+ve tumors displayed increased b-lymphocytes whereas the triple negative tumors had decreased macrophages and lymphatic invasion (131). The literature on AR phosphorylation in breast cancer is very limited; however, the post-translation status in conjunction with localization of AR deserves more research as studies suggest it may be a novel indicator of breast cancer progression in certain subtypes.

### Membranous AR as a mediator of non-genomic signaling

As alluded to above, understanding the unique locations of the AR and the functional significance of this is imperative to better understanding of its complex role in breast cancer. There is increasing evidence to suggest the existence of membrane ARs however their exact mechanism and structure has still to be elucidated. Debate surrounds whether sex steroid receptors can be located in the cell membrane or localize at the cytoplasmic membrane. Some studies suggest that membrane AR is the classical AR receptor but modifications such as palmitoylation enables translocation to the membrane (132). Alternatively, it is also possible that the mechanism is completely independent of classical AR as membrane androgen actions have been shown to occur in; cells which do not possess classical AR signaling (115), some effects appear to be mediated through G Protein Coupled Receptors (GPCR) as demonstrated by experiments using pertussis toxin inhibition (133), N-terminal AR antibodies detect membrane AR whereas C-terminal ones don't, suggesting structural differences in the receptors (133), and furthermore AR antagonists do not prevent membrane androgen responses (108, 117).

Membrane androgen sites have been documented in sertoli cells (134), osteoblasts (135), and in prostate (136), breast (137), and colon cancers (138). The Castanas group has led the field in understanding the role of membrane AR in breast cancer. They have reported on mediation of gene transcription by conjugated testosterone (that does not cross the cell membrane) and non-conjugated ligand in breast cancer. They found that in an AR negative cell line a large proportion of the genes modified by testosterone were also affected by the conjugated form of testosterone, however in AR positive cells a significant number of genes were induced with testosterone that were not observed with conjugated ligand treatment. Another interesting discovery in this study was the differential cellular pathways affected by membrane impermeable testosterone and unconjugated testosterone. Growth factor related pathways dominated the testosterone treated sample, however, inflammatory, and adhesion pathways were induced by BSA conjugated testosterone (139). A similar study found that the levels of steroids present may dictate the signaling pathway induced by membrane AR. Kampa et al. reported varying the concentration of estrogens and androgens can reverse androgen induced apoptosis and estrogen related anti-apoptotic effects (133). It has also been reported that low levels of androgens can initiate membrane-bound receptor signaling indicating that the levels of ligand present may have a direct influence on intracellular signaling (140).

Many types of G-protein coupled receptors (GPCRs) have been implication as membrane ARs. A member of the zinc ion-regulated transporter subfamily ZIP9 (SLC39A90) has been found to exhibit very similar characteristics to that of a membrane AR. The researchers propose that ZIP9 is the receptor through which testosterone induces apoptotic actions (136). Also the G –protein –coupled receptor family C group 6 member A (GPRC6A) has been shown to mediate androgen actions independent of DNA binding and to mediate testosterone induced ERK phosphorylation in prostate cancer cells (109). Kalyvianaki et al. identified the GPCR oxoeicosanoid receptor 1 (OXER1) as a specific membrane receptor that mediates rapid effects of androgens in prostate cancer cells. Testosterone binds to the same place as the natural ligand for the receptor and therefore acts as an antagonist affecting cell migration and metastasis (141). This is an active area of research and understanding and modulating the activity of membrane AR may be a key component in determining full clinical response to anti-AR therapies in subsets of breast cancer patients.

## Tumor promoting functions of AR

### The role of AR in tumor stemness and migration

There has been emerging evidence in the literature to suggest AR may play a role in regulating less differentiated more stem like cell populations (142–144). AR supports anchorage independent growth in triple negative breast cancer (145). Furthermore, in a recent study looking at the role of AR in breast cancer and its expression in circulating tumor cells (CTC) and bone metastasis, the authors report that AR is both transcribed and active in breast cancer to bone metastasis. Interestingly, on further expression analysis in CTCs they observed no change in the expression of ERα or PR in bone metastasis compared to visceral metastasis (146). Another study has also reported on the utility of AR expression on CTC's as a clinical biomarker using a platform that takes a non-biased approach. They found that patients with CTC which are AR+ve have a more heterogeneous disease (147). In line with these studies AR may also facilitate migration of cancer cells. This may involve non genomic actions as has been demonstrated in prostate cancer through AR filamin A association (148) and stromal cells (121). The protein CXCL12 and its receptor CXCR4 has been associated with breast cancer migration. Testosterone treatment induces both CXCL12 and CXCR4 expression in ER+ve breast cancer cells but only if AR and SRC1 are co-expressed. The authors suggest this may be through AR binding to an ARE on the CXCL12 promoter which leads to increased motility (149). It is well known that loss of E-cadherin is associated with cancer metastasis. A study by Liu et al. identified AR as a repressor of the E-cadherin gene in both metastatic and non-metastatic breast cancer. They found that AR activated by DHT causes a downregulation of E-cadherin which leads to metastasis in mice. This was supported by patient data showing high nuclei AR expression with low E-cadherin expression in patient with invasive breast cancer (150). As AR expression is retained in a number of metastatic breast cancers understanding the functional implication of its expression at different stages of the disease could provide the opportunity for more effective use of anti-AR stage tailored therapies (151, 152).

### Androgens and AR in visceral fat

**Figure 5:**
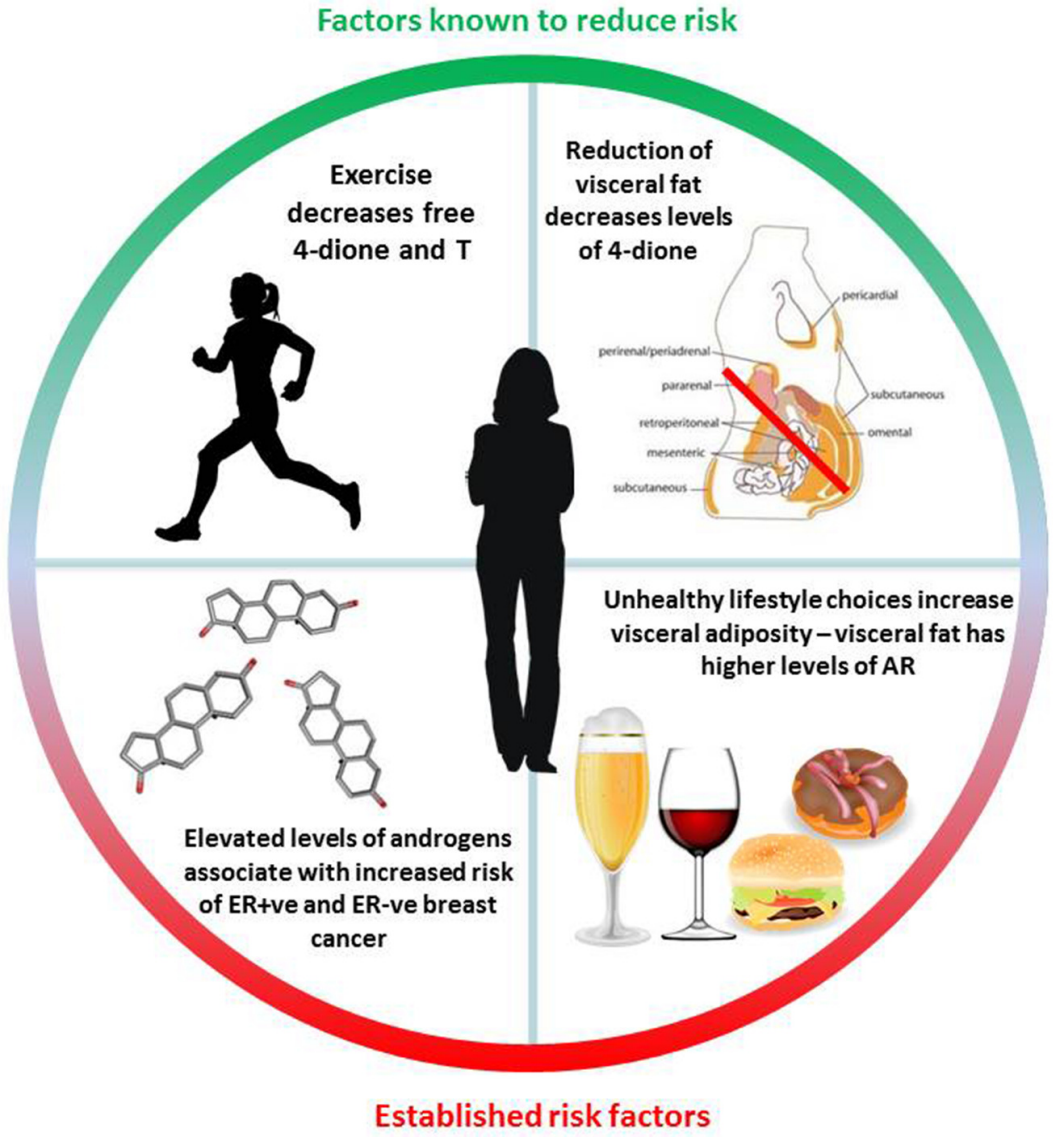
Summary of known diet and lifestyle factors associated with either increased or decreased breast cancer risk. Exercise, is particularly notable, as it is associated with a reduction in the levels of circulating androgens irrespective of weight-loss. Steroid structure Source: https://pubchem.ncbi.nlm.nih.gov/. Description: Data deposited in or computed by PubChem. Visceral adipose image source: Cook A, Cowan C. Adipose. 2009 Mar 31. In: StemBook [Internet]. Cambridge (MA): Harvard Stem Cell Institute; 2008. Figure 1, White adipose distribution in the body. Available from: https://www.ncbi.nlm.nih.gov/books/NBK27053/figure/adipose.F1/ doi: 10.3824/stembook.1.40.1.

It is now firmly established that diet and lifestyle have a major impact on the risk of developing metabolic disorders associated with an increased likelihood of developing various cancers and type 2 diabetes (153). Breast cancer risk demonstrates one of the strongest associations with obesity and in particular increased levels of visceral fat (154, 155). A number of studies have evaluated the presence of steroid receptors within adipose deposits with an emphasis on elucidating the differences between subcutaneous fat which is relatively indolent, in contrast to visceral fat which is far more metabolically active and associated with metabolic disarray. It was found that visceral fat contains much higher levels of AR and GR, displays a greater capacity to generate free fatty-acids and exhibits insulin resistance (156). Administration of exogenous androgens has been reported to modulate adipose deposition in postmenopausal women, resulting in greater visceral adiposity (157). Of note, a study that investigated abdominal fat distribution in breast cancer patients undergoing AI therapy showed a greater visceral:subcutaneous distribution which was irrespective of weight gain or loss, suggesting that the androgenic steroid environment may play a key role in driving this alteration (158). It is also established that exercise affects steroid metabolism within adipocytes and promotes anti-inflammatory adipokine secretion, reduces anti-inflammatory cytokine release and may also play an important role insulin sensitivity [reviewed (159)]. Whilst there is no evidence that tumor levels of AR protein are associated with a high body mass index (160), there is ample epidemiological evidence that exercise decreases levels of sex hormones, 4-dione, DHEA-S and markers of adiposity irrespective of weight loss (4, 160, 161). It is therefore crucial to understand the role of adipose tissue including its localization and in particular any perturbations in metabolism of prohormones such as 4-dione. Steroid levels are known to be altered by diet, exercise and other lifestyle choices; understanding their potential role in breast cancer and resistance to therapy could therefore have wide-ranging implications for prevention and survivorship (summarized in Figure 5).

## Summary

In clinical cohorts heterogeneity of AR expression combined with ERα status appears to be the criteria influencing prognostic and predictive roles of AR in breast cancer (162). However, as receptor positivity is established as staining >1%, it is important to note that different levels and location of receptors within cells may disclose more information for clinical actions and diagnosis. On a translational level the recent publication establishing a threshold level for AR positivity is likely to greatly improve clinical parameters associated with AR in breast cancer (44). In a case-control study nested within the EPIC cohort (European Prospective Investigation into Cancer and Nutrition), both serum androgens as well as estrogens were both found to be associated with risks of both hormone receptor-negative as well as receptor-positive breast tumors. The authors concluded that further research is needed to establish the molecular pathways and evolutionary stages of development, through which both androgens and estrogens can promote the occurrence of both receptor-positive and negative clinical breast tumors (24). Counter-intuitively, breast cancers have been successfully treated with either high dose estrogens or high-dose androgens; highlighting the junus-like function of both steroids (163–165). Although numerous studies have correlated circulating steroid levels with breast cancer risk it is essential that individual tumor intracrinology is evaluated to get a clear understanding of the steroid tumor microenvironment for each patient. Whilst this area has always been of interest, it was hampered by inadequate quantitative techniques. Thankfully this area of research has evolved very rapidly over the past number of years and in breast cancer the work of the Sasano group has proved to be hugely innovative (30). We now know that there is a high degree of intratumoral heterogeneity amongst patients and it will be of interest to understand if these correlate with not only patient outcome but also other factors known to impact steroid levels such as diet, adiposity, and exercise.

Overall, studies indicate that anti-AR therapy will only be an effective treatment in the presence of activated tumor promoting AR, therefore it is imperative that we understand the mechanisms of this activation in order to inform patient selection.

## Concluding remarks

A major hurdle in therapeutically targeting AR in breast cancer is that despite all the *in vitro* and clinical studies conducted we are still missing an essential part of the molecular landscape of breast cancer; that is, what dictates pro or anti tumorigenic responses to androgens. This review highlights the need to assess the role of steroid receptors in breast cancer coupled with knowledge of steroid intracrinology.

## Ethics statement

Ethical approval was granted by CTI (CTI 07/09).

## Author contributions

All authors listed have made a substantial, direct and intellectual contribution to the work, and approved it for publication.

### Conflict of interest statement

The authors declare that the research was conducted in the absence of any commercial or financial relationships that could be construed as a potential conflict of interest.
